# Supplementary material for: Chromothripsis during telomere crisis is independent of NHEJ, and consistent with a replicative origin
Source: Genome Res. 2019 May;29(5):737–49. doi: 10.1101/gr.240705.118 (PMC6499312; doi:10.1101/gr.240705.118)
Supplement: Supplemental Material [file supp_gr.240705.118_Supplemental_file_1.zip › contigs/annotated_contigs/DB77/contig.2.DB77_length_270_mean_cov_5.0.docx]

**DB77_length_270_mean_cov_5.0**

```
GATTTGACTGGGCAGTCAGGCACTCACTCACACAGGGCAGGTGGGGCTGGCTGTCAGCTTGTAGCTCATCTAGGGCTGTTGTCTGAGCG
 >chr8:953654-953828 + E=1e-93
CCTGGATTCTCTCTCATGAGACCTGTCCAGCACCTTGGTCCCCCCATACCCCAGAATGGTGGCTCGGCCCTAAGTGGGAACCAT|T|AT
                                                                                     >c
TATTTGAAATAAATGGAACATTTATAAAAAATATTTTCCCTTGGTTTAGGAGTTTTTATAGCTGAAGCTTTGTGAGAATGAGATTTGAA
hr8:5552578-5552675 + E=4e-44
GTGTG
```
